# Evidence for HIV-1 cure after *CCR5*Δ32/Δ32 allogeneic haemopoietic stem-cell transplantation 30 months post analytical treatment interruption: a case report

**DOI:** 10.1016/S2352-3018(20)30069-2

**Published:** 2020-03-10

**Authors:** Ravindra Kumar Gupta, Dimitra Peppa, Alison L Hill, Cristina Gálvez, Maria Salgado, Matthew Pace, Laura E McCoy, Sarah A Griffith, John Thornhill, Aljawharah Alrubayyi, Laura E P Huyveneers, Eleni Nastouli, Paul Grant, Simon G Edwards, Andrew J Innes, John Frater, Monique Nijhuis, Anne Marie J Wensing, Javier Martinez-Picado, Eduardo Olavarria

**Affiliations:** a Department of Medicine, University of Cambridge, Cambridge, UK; b Africa Health Research Institute, Durban, South Africa; c Nuffield Department of Medicine, University of Oxford, Oxford, UK; d Department for Organismic and Evolutionary Biology, Harvard University, Cambridge, MA, USA; e IrsiCaixa AIDS Research Institute, Badalona, Spain; f Autonomous University of Barcelona, Cerdanyola del Vallès, Spain; g Division of Infection and Immunity, University College London (UCL), London, UK; h Imperial College London, London, UK; i Department of Medical Microbiology, University Medical Center, Utrecht, Netherlands; j Department of Virology, UCL Hospitals, London, UK; k Population, Policy and Practice, UCL Great Ormond Street Institute of Child Health, London, UK; l Mortimer Market Centre, Department of HIV, Central and North West London NHS Trust, London, UK; m Imperial College NHS Healthcare Trust, Hammersmith Hospital, London, UK; n Oxford National Institute for Health Research Biomedical Research Centre, Oxford, UK; o University of Vic – Central University of Catalonia, Vic, Spain; p Catalan Institution for Research and Advanced Studies, Barcelona, Spain

## Abstract

**Background:**

The London patient (participant 36 in the IciStem cohort) underwent allogeneic stem-cell transplantation with cells that did not express CCR5 (*CCR5*Δ32/Δ32); remission was reported at 18 months after analytical treatment interruption (ATI). Here, we present longer term data for this patient (up to 30 months after ATI), including sampling from diverse HIV-1 reservoir sites.

**Methods:**

We used ultrasensitive viral load assays of plasma, semen, and cerebrospinal fluid (CSF) samples to detect HIV-1 RNA. In gut biopsy samples and lymph-node tissue, cell-copy number and total HIV-1 DNA levels were quantified in multiple replicates, using droplet digital PCR (ddPCR) and quantitative real-time PCR. We also analysed the presence of intact proviral DNA using multiplex ddPCR targeting the packaging signal (ψ) and envelope (*env*). We did intracellular cytokine staining to measure HIV-1-specific T-cell responses. We used low-sensitive and low-avidity antibody assays to measure the humoral response to HIV-1. We predicted the probability of rebound using a mathematical model and inference approach.

**Findings:**

HIV-1 viral load in plasma remained undetectable in the London patient up to 30 months (last tested on March 4, 2020), using an assay with a detection limit of 1 copy per mL. The patient's CD4 count was 430 cells per μL (23·5% of total T cells) at 28 months. A very low-level positive signal for HIV-1 DNA was recorded in peripheral CD4 memory cells at 28 months. The viral load in semen was undetectable in both plasma (lower limit of detection [LLD] <12 copies per mL) and cells (LLD 10 copies per 10^6^ cells) at 21 months. CSF was within normal parameters at 25 months, with HIV-1 RNA below the detection limit (LLD 1 copy per mL). HIV-1 DNA by ddPCR was negative in rectum, caecum, and sigmoid colon and terminal ileum tissue samples at 22 months. Lymph-node tissue from axilla was positive for the long-terminal repeat (33 copies per 10^6^ cells) and *env* (26·1 copies per 10^6^ cells), negative for ψ and integrase, and negative by the intact proviral DNA assay, at 27 months. HIV-1-specific CD4 and CD8 T-cell responses have remained absent at 27 months. Low-avidity Env antibodies have continued to decline. Mathematical modelling suggests that the probability of remission for life (cure) is 98% in the context of 80% donor chimerism in total HIV target cells and greater than 99% probability of remission for life with 90% donor chimerism.

**Interpretation:**

The London patient has been in HIV-1 remission for 30 months with no detectable replication-competent virus in blood, CSF, intestinal tissue, or lymphoid tissue. Donor chimerism has been maintained at 99% in peripheral T cells. We propose that these findings represent HIV-1 cure.

**Funding:**

Wellcome Trust and amfAR (American Foundation for AIDS Research).

## Introduction

HIV cure, exemplified by extended periods off antiretroviral therapy (ART) with negative results on testing for HIV nucleic acid and loss of adaptive immune responses, has been elusive. For a decade, only one such case, referred to as the Berlin patient, was recognised.^1^, ^2^, ^3^ This individual underwent two rounds of total body irradiation and allogeneic haemopoietic stem-cell transplantation (allo-HSCT) with donor cells that did not express CCR5 (*CCR5*Δ32/Δ32) for treatment of acute myelogenous leukaemia. In 2019 we reported HIV-1 remission in the London patient (participant 36 in the IciStem cohort [IciS-36]),^4^ who underwent one HSCT procedure with *CCR5*Δ32/Δ32 donor cells for treatment of refractory Hodgkin lymphoma (stage IVb). Remission in the London patient, shown by testing of plasma and circulating peripheral CD4 T cells for traces of HIV-1, was reported 18 months after analytical treatment interruption (ATI) of ART. Here, we present longer term clinical, laboratory, and mathematical modelling data to make the case for long-term remission of HIV-1 (cure) in this individual.

**Research in context**

**Evidence before this study**

We searched PubMed on Feb 1, 2020, with the terms “allogeneic transplantation”, “CCR5”, and “HIV”, and either “cure” or “remission”. We did not restrict our search by date or language. To date, the only reported case of long-term remission (cure) of HIV is the Berlin patient, who underwent two allogeneic haemopoietic stem-cell transplantation (allo-HSCT) procedures using cells from a homozygous *CCR5*Δ32 (*CCR5*Δ32/Δ32) donor. Despite reports showing unsuccessful allo-HSCT with *CCR5*Δ32/Δ32 tissue, in 2019 we reported an individual (the London patient) who achieved long-term suppression of HIV-1 after such a procedure. We did not report data for HIV-1 testing of reservoirs other than blood.

**Added value of this study**

We have shown in the London patient an absence of HIV-1 replication in samples of blood, cerebrospinal fluid, semen, intestinal tissue, and lymphoid tissue up to 30 months after analytical treatment interruption of antiretroviral therapy. Remnants of integrated HIV-1 DNA sequences that are unlikely to be capable of producing virus remain in tissue samples and can be regarded as so-called fossils. Antibodies to HIV-1 envelope protein remain positive.

**Implications of all the available evidence**

Long-term remission of HIV-1 can be achieved using reduced-intensity drug regimens with one *CCR5*Δ32/Δ32 allo-HSCT procedure without total body irradiation. Evidence of past HIV infection might persist analogous to antibody responses to other viral infections that have been cleared.

## Methods

### Viral load testing

All testing was done at University College Hospitals NHS Trust (London, UK). We used standard clinically validated hospital laboratory assays to measure HIV-1 viral load in plasma and semen samples. Further, we used an in-house ultrasensitive plasma and cerebrospinal fluid (CSF) viral load assay to detect HIV-1 RNA. Seminal plasma was separated from the cellular fraction by centrifugation. We centrifuged 8 mL plasma or CSF at 21 000 g for 2 h at 4°C before removing the supernatant and resuspending the pellet in 700 μL of residual plasma. We then tested the suspension using the Hologic Aptima HIV-1 Quant Dx assay (Marlborough, MA, USA). We measured whole leucocyte and T-cell-specific (CD3-selected) chimerism by short tandem repeat analysis with the PowerPlex16 system (Promega, Madison, WI, USA).

### Blood and tissue processing

We isolated peripheral blood mononuclear cells (PBMCs) from 60 mL EDTA (edetic acid) blood by centrifugation on a Ficoll gradient, and did magnetic activated cell sorting to retrieve naive T cells and memory T cells (CD4^+^ T-Cell and Tnaïve CD4^+^ Cell Isolation Kit; Miltenyi Biotec, Cologne, Germany). DNA was isolated using a DNeasy Blood and Tissue kit (Qiagen, Hilden, Germany). Biopsy samples of gut tissue (rectum, caecum, and sigmoid colon and terminal ileum) were obtained by colonoscopy and homogenised using ceramic beads in a MagNA Lyser (Roche, Basel, Switzerland) set at 6000 rpm for 45 s, as described.^5^ DNA was extracted using an Qiagen AllPrep DNA/RNA Mini kit. Increased uptake of ^18^F-fluorodeoxyglucose in an axillary lymph node was seen on PET-CT and a diagnostic lymph-node biopsy sample was taken. The biopsy specimen was processed the same way as were the gut biopsy samples.

### Quantitative real-time PCR

For each quantitative real-time PCR (rtPCR), standards were tested in triplicate, in addition to two positive control wells in triplicate and six negative control wells. We used 25 000 cells' worth of DNA (based on albumin rtPCR) per well for the London patient. The packaging site (ψ) rtPCR amplifies a fragment between positions 692 and 797. The envelope (*env*) PCR amplifies a fragment between positions 7736 and 7851.

### Droplet digital PCR

We quantified HIV-1 DNA using droplet digital PCR (ddPCR; Bio-Rad, Hercules, CA, USA) targeting the long terminal repeat (LTR), gag, and integrase regions, shown as target copies per 10^6^ cells tested.^6^ We measured in duplicate amounts of the human gene for RNase P (*RPP30*), which is present twice in diploid cells, to ascertain the input cell number. In all ddPCR runs, water and donor PBMCs were tested in duplicate as negative controls for HIV target regions and U1 cells were tested as a positive control. Samples generating one positive droplet were interpreted as negative based on the occurrence of a sporadic positive droplet in the negative controls (one in 40 reactions). We further analysed samples showing at least two positive droplets in an intact proviral DNA assay (IPDA), essentially as described.^7^ The IPDA assay is based on a duplex ddPCR targeting two regions in the viral genome that are present in most intact proviruses, ψ and the Rev response element (RRE) in *env*. The *env* ddPCR also contains a probe without a fluorescent label that can discriminate G-A hypermutated proviruses. Again, to ascertain cell number and DNA shearing, *RPP30* was targeted and ddPCR controls were used. The DNA shearing index was calculated to correct the number of intact proviruses (double positives for ψ and *env*).

### HIV-1, cytomegalovirus, and Epstein-Barr virus specific CD4 and CD8 T-cell responses

For intracellular cytokine staining and peptide stimulation, PBMCs were thawed and resuspended in RPMI complete media. After overnight rest at 37°C and 5% carbon dioxide, PBMCs were stimulated for 6 h with 2 μg/mL HIV-1 Gag pools or cytomegalovirus (CMV) pp65 (JPT Peptide Technologies, Berlin, Germany) or the PepTivator Epstein-Barr virus (EBV) consensus pool (Miltenyi Biotec) containing 43 peptides of between eight and 20 amino acids in length from the proteins LMP2A, BRLF1, BMLF1 (EB2), BNLF1 (LMP1), BERF3 (EBNA6), BERF1 (EBNA3), BERF2 (EBNA4), BALF2 (DNBI), BMRF1, BZLF1, BNRF1 (MTP), EBNA1, BLLF1 (gp350), and BARF in the presence of 1 μg/mL anti-CD28 and anti-CD49d CoStim antibodies (BD Biosciences, Cowley, UK), 2 μmol/L Monensin (BD Biosciences), 10 μg/mL Brefeldin A (Sigma, St Louis, MO, USA), and anti-CD107a APC-H7 antibody (BD Biosciences). After stimulation, virus-specific T cells were identified by intracellular cytokine staining.^4^ In brief, cells were surface stained with antibodies (CD14 BV510, CD19 BV510, CD3 BV650, CD4 BV711, CD8 BV421; Biolegend, San Diego, CA, USA) in the presence of fixable Live/Dead stain (Invitrogen, Waltham, MA, USA). Cells were then fixed and permeabilised (CytoFix/CytoPerm; BD Biosciences) followed by intracellular cytokine staining for interferon γ with PE-Cyanine7, tumour necrosis factor with fluorescein isothiocyanate, and interleukin 2 with PercP eFluor710 (eBioscience, Waltham, MA, USA). Stimulation with 0·005% dimethyl sulphoxide in the presence of costimulatory antibodies, protein transport inhibitors, and CD107a was done as a negative control. Samples were acquired on a BD Fortessa X20 using BD FACSDiva8.0 (BD Biosciences). Data were analysed using FlowJo 10 (BD, Ashland, OR, USA).

### HIV-1 antibody responses

Specific HIV-1 antibodies in longitudinal serum samples diluted one part in two parts were tested in a qualitative western blot assay (New Lav Blot I; Bio-Rad). Standard and low-sensitive versions of the Vitros anti-HIV-1 assay (Ortho-Clinical Diagnostics, High Wycombe, UK) and the limiting avidity antigen assay were measured in the same samples as described.^6^ Briefly, four recombinant antigens (HIV-1 Env 13, HIV-1 Env 10, HIV-1 p24, and HIV-2 Env AL) derived from HIV-1 core, HIV-1 Env, and HIV-2 Env proteins were quantified. The optimised version of the low-sensitive Vitros assay uses a one part in 400 parts dilution of the HIV-positive sample. The cutoff was set at a signal to cut-off ratio (S/CO) of 20.^6^ The avidity assay measures the capacity of guanidine to elute low-avidity and low-affinity antibodies after antigen–antibody bonds have formed. The results are reported as an avidity index, calculated as the ratio of the S/CO of the sample incubated in guanidine to the S/CO of the sample incubated in PBS, with 0·51 set as the cutoff.^7^

### Mathematical modelling

The mathematical modelling analysis consisted of two parts. First, we used a mathematical model^8^ to simulate the expected distribution of rebound times as a function of reservoir size and target cell fraction (ie, chimerism). Second, we used this model with a previously developed Bayesian inference framework^9^, ^10^ to interpret the outcome of the London patient. In particular, we estimated the posterior probability of a particular number of cells remaining in the latent reservoir, in view of both the results of laboratory assays and the observation of no rebound for a specific time off ART, and the likelihood that the patient will have a rebound sometime in the future (*vs* lifetime remission) as a function of both the degree of target cell susceptibility (*CCR5*Δ32/Δ32 chimerism) and the current time off ART without rebound (appendix pp 1–5).

### Ethics and consent

The London patient was registered to the IciStem consortium as IciS-36. We obtained ethics approval from the UK National Health Service Health Research Authority Research Ethics Committee in April, 2017 (reference 17/SW/0021, protocol no 16/0594). The patient provided full written informed consent in July, 2017. The patient also reviewed the final version of the updated Article and provided written consent to its publication.

### Role of the funding source

The funder had no role in study design, data collection, data analysis, data interpretation, or writing of the report. The corresponding author had full access to all data in the study and had final responsibility for the decision to submit for publication.

## Results

**Figure 1:**
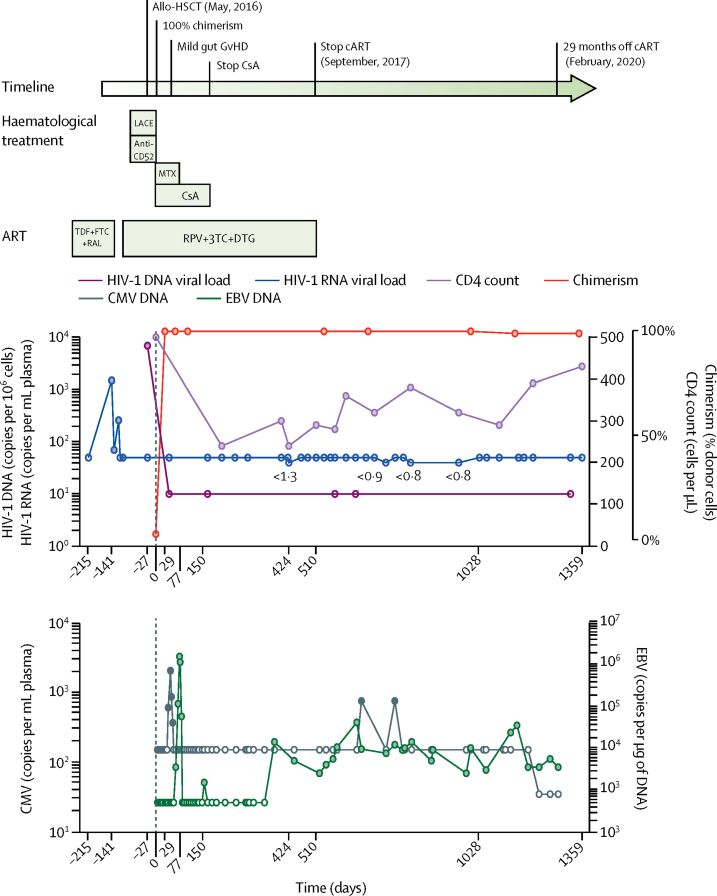
Clinical course of the London patient up to 29 months after analytical treatment interruption Upper panel shows peripheral blood CD4 count, plasma HIV-1 RNA, HIV-1 DNA, and chimerism in peripheral T cells over time. Lower panel shows amounts in DNA of CMV and EBV in plasma over time. Anti-CD52 was alemtuzumab. 3TC=lamivudine. Allo-HSCT=allogeneic haemopoietic stem-cell transplantation. cART=combination antiretroviral therapy. CMV=cytomegalovirus. CsA=ciclosporin. DTG=dolutegravir. EBV=Epstein-Barr virus. GvHD=graft versus host disease. FTC=emtricitabine. LACE=lomustine, cytarabine, cyclophosphamide, and etoposide. MTX=methotrexate. RAL=raltegravir. RPV=rilpivirine. TDF=tenofovir disoproxil fumarate.

Since 2019,^4^ the London patient has shown somewhat slow CD4 reconstitution (figure 1). At 28 months after ATI, the CD4 count reached near pretransplant levels (430 cells per μL [23·5% of total T cells]), with CD4:CD8 of 0·86 (normal range 1·5–2·5). However, no opportunistic infections were reported and peripheral T-cell chimerism was maintained at 99% (figure 1). HIV-1 plasma viral load was monitored up to 30 months after ATI, using a hospital-based assay with a lower limit of detection (LLD) of 20 copies per mL, and viral load was below the LLD (<1 copy per mL) up to 30 months after ATI using an ultrasensitive assay (viral load on March 4, 2020, <1 copy per mL). Reactivation of EBV at around 21 months after ATI, with peak viral load of 35 000 copies per mL plasma, was managed conservatively without specific treatment and viral load fell to 3500 copies per mL at 23 months. No clinically significant CMV reactivation was seen from 18 months to 28 months post ATI (figure 1). Of note, no further episodes of graft versus host disease (GvHD) were reported since gut GvHD at 2 months post-transplant,^4^ and no immunosuppressive drugs were taken after ATI. The London patient did not use ART for pre-exposure prophylaxis.

HIV-1 LTR ddPCR analyses were negative in bulk CD4 T cells (<2·4 LTR copies per 10^6^ cells) and in the naive CD4 T-cell subset (<3·5 LTR copies per 10^6^ cells) 28 months after ATI. In the memory subset, two positive droplets were detected in the LTR ddPCR in one of the eight replicates. IPDA showed one droplet positive in six replicates for both ψ and HIV *env*. HIV *env* was also detected as one positive droplet in two other wells (appendix p 7). The DNA shearing index was 0·24. No correction for shearing was applied in view of the uncertainty of a false-positive signal with one double-positive droplet.

A semen sample was obtained from the individual at 21 months after ATI. Semen viraemia was below the LLD in both seminal plasma (<12 copies per mL) and cells (<10 copies per 10^6^ cells). It was not possible to quantify the input number of cells in this assay, however.

Because of neurological symptoms, the London patient underwent MRI and lumbar puncture 25 months after ATI. CSF contained normal protein with no cells (<5 cells per μL) detected. HIV-1 viral load in CSF was negative (LLD 1 copy per mL).

Colonoscopy was done 22 months after ATI and tissue biopsy samples were taken. No evidence was seen of recurrence of gut GvHD. HIV-1 LTR and integrase ddPCR analyses were negative in all gut biopsy specimens (sigmoid colon and ileum >0·2 million cells tested; caecum >0·3 million cells tested; rectum >0·15 million cells tested; appendix p 8). HIV-1 LTR DNA by rtPCR was also negative in rectum, caecum, and sigmoid colon and terminal ileum (appendix p 9).

The London patient had increased metabolic activity in an axillary lymph node on PET-CT and underwent diagnostic lymph node biopsy 27 months after ATI. Histology was reported as showing follicular hyperplasia with follicle lysis and large numbers of EBV-positive cells within the follicles, most likely a reactive lymphadenopathy. The lymph node was positive for HIV-1 *env* (26·1 copies per 10^6^ cells) and LTR (33 copies per 10^6^ cells) by ddPCR, whereas the gag ddPCR was only slightly positive (5·1 copies per 10^6^ cells) and no integrase DNA could be detected (<0·9 cells per 10^6^ cells; appendix p 6). In line with these observations, no intact proviral DNA was recorded by the IPDA assay (<0·5 intact proviral DNA copies per 10^6^ cells). The DNA shearing index was 0·39. Correction for shearing could not be applied in the absence of double-positive droplets.

In a second assay that used rtPCR for detection of HIV-1 DNA in lymph-node cells, a positive low-level *env* signal was identified at around 70 copies per 10^6^ cells and a positive low-level signal was seen in ψ at around 76 copies per 10^6^ cells (appendix p 9), though IPDA was negative. HIV-1 LTR detection by rtPCR was negative in all wells. Both positive controls in triplicate on two plates were positive and all 12 negative controls were negative. Sequencing using HIV-1 *env* specific primers was unsuccessful.

**Figure 2:**
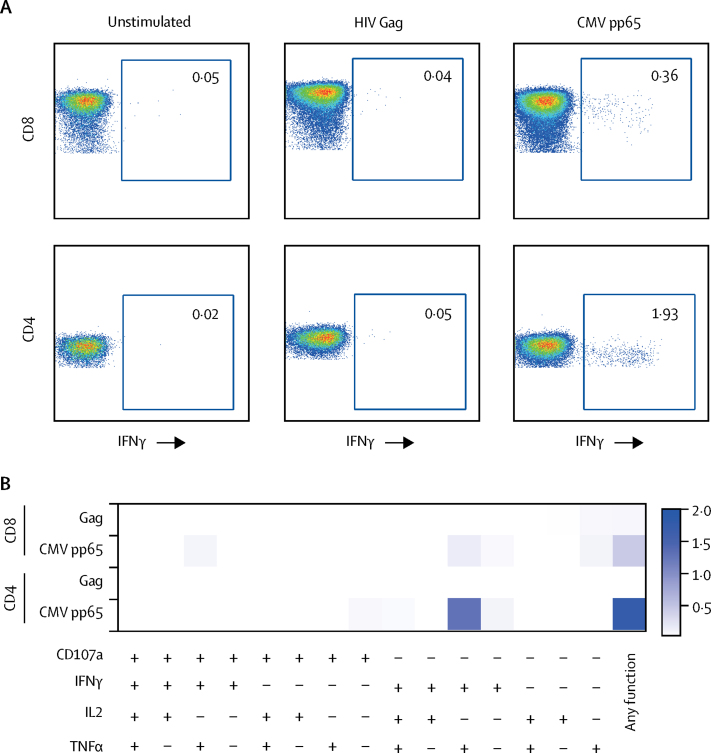
HIV Gag-specific and CMV-specific T-cell responses Representative fluorescence-activated cell sorting plots showing percentage of virus-specific CD8 T cells (upper panel) and CD4 T cells (lower panel) identified via intracellular staining for IFNγ after stimulation with HIV Gag or CMV pp65 peptide pools at 1309 days after allo-HSCT. A negative control containing peripheral blood mononuclear cells from the London patient but without peptide mix was included (unstimulated) for each assay (A). Polyfunctional profile for CD8 and CD4 T-cell responses to CMV pp65 and HIV Gag peptide stimulation subsequent to Boolean gating. The functions are listed along the *x* axis with each of their respective combinations (B). Allo-HSCT=allogeneic haemopoietic stem-cell transplantation. CMV=cytomegalovirus. IFNγ=interferon γ. IL2=interleukin 2. TNFα=tumour necrosis factor α.

**Figure 3:**
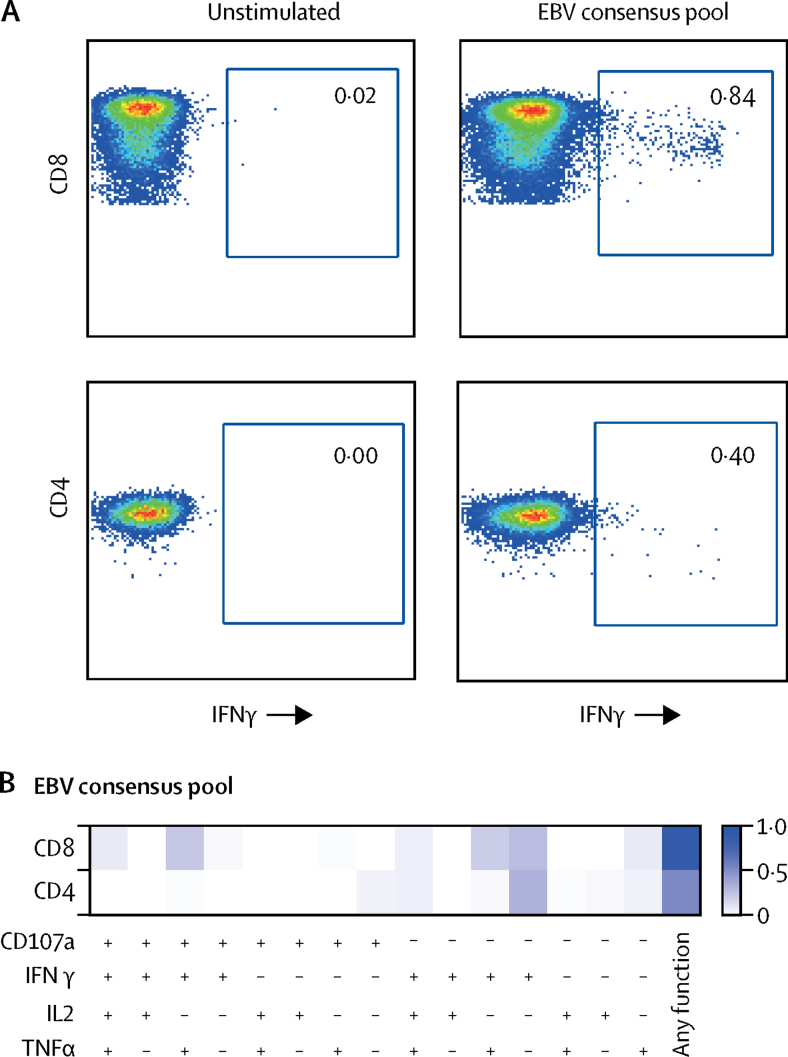
EBV-specific T-cell responses Representative fluorescence-activated cell sorting plots showing percentage of virus-specific CD8 T cells (upper panel) and CD4 T cells (lower panel) identified via intracellular staining for IFNγ after stimulation with PepTivator EBV consensus pool at 1309 days after allo-HSCT (A). A negative control containing peripheral blood mononuclear cells from the London patient but without peptide mix was included (unstimulated) for each assay (A). Polyfunctional profile for CD8 and CD4 T-cell responses to EBV peptide pool stimulation subsequent to Boolean gating. The functions are listed along the *x* axis with each of their respective combinations (B). Allo-HSCT=allogeneic haemopoietic stem-cell transplantation. EBV=Epstein-Barr virus. IFNγ=interferon γ. IL2=interleukin 2. TNFα=tumour necrosis factor α.

The London patient had previously shown immune responses against CMV, but not HIV-1, up to the 18-month timepoint.^4^ We measured CD8 and CD4 T-cell virus-specific responses after stimulation with HIV-1 Gag and CMV pp65 overlapping peptide pools. At 27 months after ATI, no interferon γ responses or polyfunctional responses were noted to HIV-1 Gag in CD4 and CD8 cells (figure 2). By contrast, CMV pp65 reactivity in CD4 and CD8 T cells was detected. In view of the lymph node histological findings showing B-cell proliferation, we wondered whether EBV reactivation could have triggered EBV-specific CD4 and CD8 T-cell responses and proliferation, potentially including CD4 T cells containing HIV-1 DNA. Therefore, we measured EBV-specific CD8 and CD4 T-cell responses in peripheral blood. CD8 responses were robust and smaller CD4 responses were also observed (figure 3).

**Figure 4:**
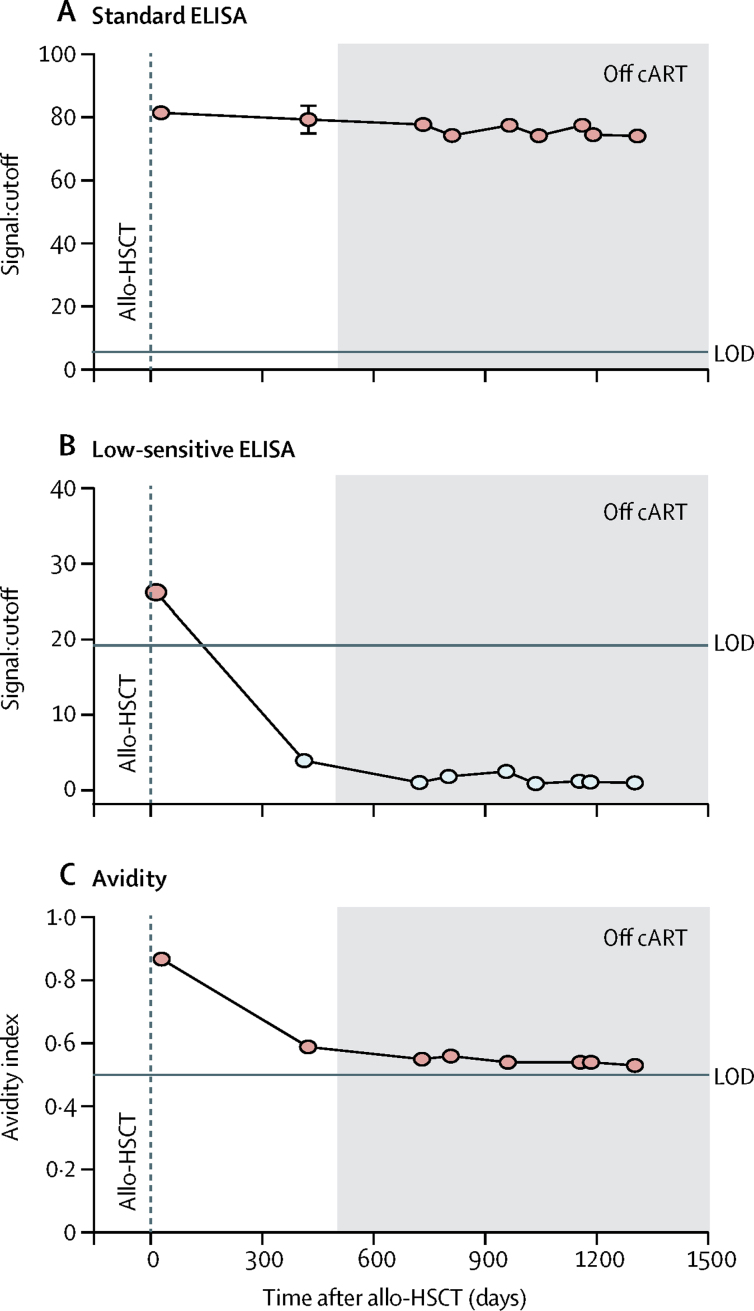
HIV-specific antibodies Humoral response dynamics were tested up to 1316 days after allo-HSCT. Antibody levels were measured using the standard HIV-1 Vitros assay (A), a detuned low-sensitive version of the HIV-1 Vitros assay (B), and the limiting antigen avidity assay (C). White circles represent values under the LOD. Grey shading denotes the period off cART. Allo-HSCT=allogeneic haemopoietic stem-cell transplantation. cART=combined antiretroviral therapy. LOD=limit of detection.

Antibodies against HIV-1 core and Env proteins measured by standard ELISA persisted within the same values more than 2 years after ATI. However, the low-sensitive Vitros analysis and antibody avidity assay showed decreased levels of HIV-1 antibodies after allo-HSCT, which were maintained after 2 years of ATI (figure 4). Western blot analysis of antibodies showed a persistent loss of multiple bands since allo-HSCT until 2 years after ATI, but with persistent presence of gp160 and gp110/120 antibodies in all samples analysed (appendix p 9).

**Figure 5:**
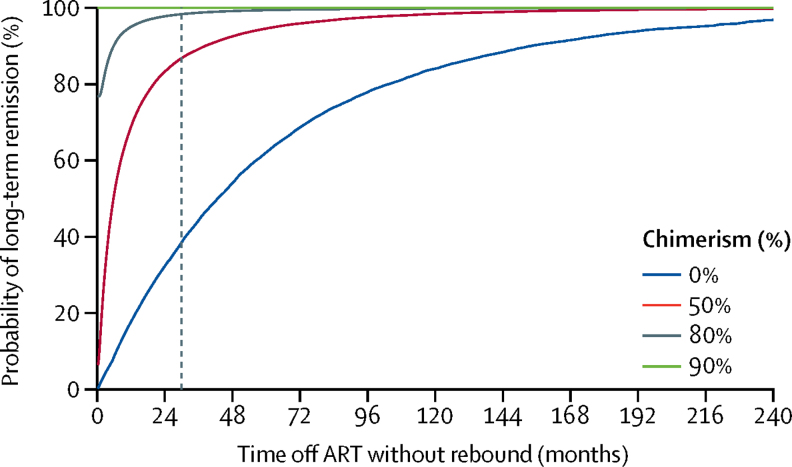
Estimated probability of long-term remission in the London patient Probability of long-term remission (cure) is calculated as a function of the observed time off ART without rebound and the fraction of target cells that are protected from infection. Calculations were based on a stochastic mathematical model of reservoir dynamics and rebound. The dotted horizontal line shows the current time off ART of 29 months. Long-term remission was defined as 70 years without rebound. ART=antiretroviral therapy.

The absence of rebound after ART interruption could be due to two mechanisms: a reduction in the latent reservoir after conditioning and transplantation;^11^, ^12^ and a reduction in the fraction of susceptible target cells for CCR5 tropic virus because of engraftment of donor *CCR5*Δ32/Δ32 cells.^13^, ^14^ To understand the relative role of each mechanism alone and in combination, we used a mathematical model of latent and active HIV-1 dynamics. This model is based on previously developed tools to understand the effect of reservoir size on rebound time^8^, ^9^, ^10^ and is adapted to account for the effect of a reduced target cell pool on viral growth (appendix pp 1–5). Although donor cell chimerism was measured in PBMCs to be approximately 99%, we postulated that this fraction could be lower in tissue sites vital for HIV-1 replication. Therefore, we considered a range of scenarios for the fraction of cells susceptible to HIV-1 infection. For each scenario, we simulated the distribution of expected rebound times and the probability of long-term remission (cure) as a function of reservoir size at the time of ART interruption (appendix p 3). These results were then used to calculate the probability that the patient would remain in remission for life, in view of observations of 29 months without rebound and no viral outgrowth seen in 24 million CD4 T cells, as previously reported at 18 months^4^ (figure 5). If target cells remain susceptible to infection (and reservoir reduction is the main mechanism for delayed rebound), the model predicts there is only a 38% chance the patient will be in remission for life. With 50% chimerism, the likelihood of full remission jumps to 87%; with 80% chimerism, the chance of remission rises to 98%. For 90% or higher reduction in susceptible cells, we estimate a negligible chance of future rebound.

## Discussion

After extensive tissue sampling completed by 3·5 years after ATI, the Berlin patient was reported as being cured from HIV-1.^3^ The London patient has been in HIV remission for 30 months (as of March 4, 2020), with plasma negative for HIV-1 RNA (<1 copy per mL) and semen negative at less than 12 copies per mL at 21 months. HIV-1 replication can occur in the CNS even during suppressive ART^15^ and is frequently associated with symptoms and MRI abnormalities.^16^ However, no detectable virus was recorded in CSF. Similarly, intestinal lymphoid tissue is an important HIV reservoir^17^ and HIV-1 DNA was negative in gut biopsy samples by ddPCR and rtPCR.

Similar to the Berlin patient,^18^ highly sensitive tests showed very low levels of so-called fossilised HIV-1 DNA in some tissue samples from the London patient. Residual HIV-1 DNA in axillary lymph node tissue could represent a defective clone that expanded during hyperplasia within the lymph node sampled. In support of this hypothesis, we recorded EBV-specific CD4 and CD8 responses in peripheral blood, although we were unable to separate lymph node-associated T cells for testing. Sequence analysis of HIV-1 DNA in lymph nodes was not successful, probably because of the very low copy number; therefore, we cannot fully rule out the possibility that very low levels of intact HIV-1 provirus have persisted. The signal could also be attributable to contamination, although sequencing would be needed to investigate this possibility further.

Myeloid cells (eg, macrophages) are also present in lymph nodes and are susceptible to both T-cell dependent and independent infection with HIV-1,^19^, ^20^, ^21^, ^22^ and they survive chemotherapy with drugs such as etoposide.^23^, ^24^ Myeloid cells can also proliferate^25^ and could, therefore, have contributed to the noted HIV-1 DNA signal in lymph node tissue.

Moreover, a low-level signal in peripheral memory CD4 T cells was recorded by ddPCR. Such occasional low-level positive signals were also seen in the Berlin patient^18^ and, previously, in CD4 T cells in the London patient,^26^ which could reflect a false ddPCR signal, potential contamination, or evidence of very low levels of persistent HIV-1 DNA-positive cells.

HIV-1-specific CD4 and CD8 T-cell responses also remained below detection, by contrast with maintenance of CMV-specific responses. We saw declining responses in HIV-1 specific antibodies up to 27 months after ATI using detuned (low-sensitive) and low-avidity assays, despite positive ELISA. Western blot results showed similar appearances at 27 months compared with at 18 months after ATI, with only gp160 antibodies remaining. These results are consistent with a positive standard ELISA against HIV-1 *env* reported previously both in the London patient and the Berlin patient.^1^, ^4^ We believe that persistent HIV-1 Env-specific plasma cells could have survived allo-HSCT^27^ and that the low-level *env* PCR signal in lymph nodes could be related to this observation. Standard ELISA testing, therefore, cannot be used as a marker for cure, although more work needs to be done to assess the role of detuned and low-avidity antibody assays in defining cure.

The slow recovery in CD4 count in the London patient could be the result of the lengthy duration of untreated HIV infection between 2003 and 2013 or to the effects of alemtuzumab (anti-CD52 monoclonal antibody used for lymphodepletion in the setting of allo-HSCT). The noted reactivation of EBV at 21 months after ATI could have been related to this continued relative immunosuppression, although CMV reactivation did not occur at that time and no opportunistic infections arose. Importantly, the London patient did not receive any cytotoxic agents or GvHD prophylaxis beyond 6 months after allo-HSCT.^4^

Mathematical modelling suggested that if more than 80% of target cells were donor-derived then remission for life (cure) was 98% likely in view of no rebound at 29 months. With chimerism higher than 90%, cure is almost certain in the model. These results can be understood along the same principle as the vaccination threshold in epidemiology.^28^ As long as a critical fraction of target cells are protected (eg, a critical percentage of chimerism is reached), the infection cannot grow exponentially. This critical fraction depends on the value of the basic reproduction ratio (R_0_) for the infection, and a distribution for this value across patients can be estimated from viral growth rates during acute infection combined with estimates of the HIV generation time (appendix pp 1–5). We plan to do viral load testing twice a year up to 60 months after ATI before testing yearly to 120 months.

In conclusion, we believe these findings probably represent the second recorded HIV-1 cure after *CCR5*Δ32/Δ32 allo-HSCT, with evidence of residual low-level HIV-1 DNA. Despite showing (in both the London patient and the Berlin patient) that CCR5-directed approaches can lead to long-term remission of HIV-1, several barriers remain to be overcome (eg, gene editing efficiency and robust safety data) before *CCR5* gene editing can be used as a scalable cure strategy for HIV-1.^29^, ^30^, ^31^

## References

[1] Hutter G, Nowak D, Mossner M (2009). Long-term control of HIV by *CCR5* Delta32/Delta32 stem-cell transplantation. N Engl J Med.

[2] Hutter G, Thiel E (2011). Allogeneic transplantation of CCR5-deficient progenitor cells in a patient with HIV infection: an update after 3 years and the search for patient no. 2. AIDS.

[3] Allers K, Hutter G, Hofmann J (2011). Evidence for the cure of HIV infection by CCR5Δ32/Δ32 stem cell transplantation. Blood.

[4] Gupta RK, Abdul-Jawad S, McCoy LE (2019). HIV-1 remission following CCR5Δ32/Δ32 haematopoietic stem-cell transplantation. Nature.

[5] Thornhill JP, Pace M, Martin GE (2019). CD32 expressing doublets in HIV-infected gut-associated lymphoid tissue are associated with a T follicular helper cell phenotype. Mucosal Immunol.

[6] Keating SM, Hanson D, Lebedeva M (2012). Lower-sensitivity and avidity modifications of the vitros anti-HIV 1+2 assay for detection of recent HIV infections and incidence estimation. J Clin Microbiol.

[7] Fernandez G, Manzardo C, Montoliu A (2015). Evaluation of an antibody avidity index method for detecting recent human immunodeficiency virus type 1 infection using an automated chemiluminescence immunoassay. Enferm Infecc Microbiol Clin.

[8] Hill AL, Rosenbloom DI, Fu F, Nowak MA, Siliciano RF (2014). Predicting the outcomes of treatment to eradicate the latent reservoir for HIV-1. Proc Natl Acad Sci USA.

[9] Hill AL, Rosenbloom DI, Goldstein E (2016). Real-time predictions of reservoir size and rebound time during antiretroviral therapy interruption trials for HIV. PLoS Pathog.

[10] Henrich TJ, Hatano H, Bacon O (2017). HIV-1 persistence following extremely early initiation of antiretroviral therapy (ART) during acute HIV-1 infection: an observational study. PLoS Med.

[11] Salgado M, Kwon M, Galvez C (2018). Mechanisms that contribute to a profound reduction of the HIV-1 reservoir after allogeneic stem cell transplant. Ann Intern Med.

[12] Henrich TJ, Hanhauser E, Marty FM (2014). Antiretroviral-free HIV-1 remission and viral rebound after allogeneic stem cell transplantation: report of 2 cases. Ann Intern Med.

[13] Kordelas L, Verheyen J, Beelen DW (2014). Shift of HIV tropism in stem-cell transplantation with *CCR5* delta32 mutation. N Engl J Med.

[14] Duarte RF, Salgado M, Sánchez-Ortega I (2015). *CCR5* Δ32 homozygous cord blood allogeneic transplantation in a patient with HIV: a case report. Lancet HIV.

[15] Collier DA, Haddow L, Brijkumar J, Moosa MS, Benjamin L, Gupta RK (2018). HIV cerebrospinal fluid escape and neurocognitive pathology in the era of combined antiretroviral therapy: what lies beneath the tip of the iceberg in sub-Saharan Africa?. Brain Sci.

[16] Kugathasan R, Collier DA, Haddow LJ (2017). Diffuse white matter signal abnormalities on magnetic resonance imaging are associated with human immunodeficiency virus type 1 viral escape in the central nervous system among patients with neurological symptoms. Clin Infect Dis.

[17] Estes JD, Kityo C, Ssali F (2017). Defining total-body AIDS-virus burden with implications for curative strategies. Nat Med.

[18] Yukl SA, Boritz E, Busch M (2013). Challenges in detecting HIV persistence during potentially curative interventions: a study of the Berlin patient. PLoS Pathog.

[19] Mlcochova P, Sutherland KA, Watters SA (2017). A G1-like state allows HIV-1 to bypass SAMHD1 restriction in macrophages. EMBO J.

[20] Watters SA, Mlcochova P, Gupta RK (2013). Macrophages: the neglected barrier to eradication. Curr Opin Infect Dis.

[21] Baxter AE, Russell RA, Duncan CJ (2014). Macrophage infection via selective capture of HIV-1-infected CD4^+^ T cells. Cell Host Microbe.

[22] Yukl SA, Sinclair E, Somsouk M (2014). A comparison of methods for measuring rectal HIV levels suggests that HIV DNA resides in cells other than CD4+ T cells, including myeloid cells. AIDS.

[23] Mlcochova P, Caswell SJ, Taylor IA, Towers GJ, Gupta RK (2018). DNA damage induced by topoisomerase inhibitors activates SAMHD1 and blocks HIV-1 infection of macrophages. EMBO J.

[24] Jauregui P, Landau NR (2018). DNA damage induces a SAMHD1-mediated block to the infection of macrophages by HIV-1. Sci Rep.

[25] Jenkins SJ, Ruckerl D, Cook PC (2011). Local macrophage proliferation, rather than recruitment from the blood, is a signature of TH2 inflammation. Science.

[26] Bruner KM, Wang Z, Simonetti FR (2019). A quantitative approach for measuring the reservoir of latent HIV-1 proviruses. Nature.

[27] Small TN, Robinson WH, Miklos DB (2009). B cells and transplantation: an educational resource. Biol Blood Marrow Transplant.

[28] Fine P, Eames K, Heymann DL (2011). “Herd immunity”: a rough guide. Clin Infect Dis.

[29] Xu L, Wang J, Liu Y (2019). CRISPR-edited stem cells in a patient with HIV and acute lymphocytic leukemia. N Engl J Med.

[30] Tebas P, Stein D, Tang WW (2014). Gene editing of *CCR5* in autologous CD4 T cells of persons infected with HIV. N Engl J Med.

[31] Ndung'u T, McCune JM, Deeks SG (2019). Why and where an HIV cure is needed and how it might be achieved. Nature.

