# Supplementary material for: Evidence for HIV-1 cure after CCR5Δ32/Δ32 allogeneic haemopoietic stem-cell transplantation 30 months post analytical treatment interruption: a case report
Source: Lancet HIV. 2020 Mar 10;7(5):e340–7. doi: 10.1016/S2352-3018(20)30069-2 (PMC7606918; doi:10.1016/S2352-3018(20)30069-2)
Supplement: Supplementary appendix [file mmc1.pdf]

THE LANCET
HIV

Supplementary appendix

This appendix formed part of the original submission and has been peer reviewed. We post it as supplied by the authors.

Supplement to: Gupta RK, Peppa D, Hill AL, et al. Evidence for HIV-1 cure after *CCR5*Δ32/Δ32 allogeneic haemopoietic stem-cell transplantation 30 months post analytical treatment interruption: a case report. *Lancet HIV* 2020; published online March 10. https://doi.org/10.1016/S2352-3018(20)30069-2.

Appendix

Supplementary Methods

Mathematical modelling

Our analysis consists of two parts. First, we use a mathematical model (based on [1]) to simulate the expected distribution of rebound times as a function of reservoir size and target cell fraction (i.e. chimerism). Then, we use this model with a previously-developed Bayesian inference framework [2,3] to interpret the outcome of the "London patient". In particular, we estimate a) the posterior probability of a particular number of cells remaining in the latent reservoir given both the results of laboratory assays and the observation of no rebound for a certain time off ART, and b) the likelihood that the patient will experience rebound sometime in the future (vs lifetime remission) as a function both the degree of target cell susceptibility (CCR5Δ32/Δ32 chimerism) and the current time off ART without rebound.

<u>Model description</u>

We use a stochastic model of latent and active HIV infection dynamics after ART interruption that was described in detail in previous work [1]. Briefly, for each individual patient, this model tracks the dynamics of each cell remaining in the latent reservoir and the probability and timing of viral rebound (defined as viral load of 200 copies/mL). We simulate the model for cohorts of 10,000 patients with a distribution of model parameter values taken from previous literature. In addition to a baseline cohort, we simulate hundreds of intervention cohorts in which all individuals receive a fixed reduction in the number of latently infected cells (e.g. due to pre-transplant conditioning and graft-vs-host disease), the pool of target cells for infection (e.g. due to engraftment of CCR5Δ32/Δ32 cells), or both. Variability in outcomes across patients predicted by the model thus comes both from the inherent stochastic dynamics of reservoir reactivation and infection growth, as well as from interpatient variability in the underlying parameter values.

We made several changes in the model from previous work [1]. Firstly, we allowed for changes in the infection kinetics of reactivated cells due to transplant-driven reductions in CCR5+ target cells. We assumed that if the donor chimerism was c (between 0 and 100%), then the fraction of target cells available to the virus (i.e. CD4+ CCR5+ T cells) was reduced by (1- c), and so that rate at which actively infected cells produced new infections was also reduced by 1- c. The basic reproductive ratio R_0 describes average number of secondary infections produced by a single actively infected cell [5], and the baseline R_0 value in a patient is reduced to $R_0(1- c)$ after transplant. If $R_0(1- c) <1$, there is no possibility of rebound. Secondly, we included an "eclipse phase" where infected cells experience a delay before actively producing virions that lead to new infections. Including this phase is necessary to accurately estimate R_0 from the early viral growth rate [5–7]. Thirdly, we assumed that a patient who has undergone HSCT will

have lost most of their HIV-specific immunity [8,9], and so we expect viral kinetics during rebound to be more similar to those during acute infection as compared to those during standard treatment interruption studies. Therefore we estimated the early viral growth rate, and hence the R_0 of infection, from studies of very early infection [7,10].

Parameter values

As in our previous work [1], composite parameters were estimated from a variety of sources and used to inform the individual model parameters. The population-level distribution for the net rate of decay of the latent reservoir during ART (the sum of cell death and reactivation) was estimated from longitudinal studies of the fraction of latent cells measured by quantitative viral outgrowth assays [11,12]. The distribution used was normal with mean 5.2×10^{-4} /day and standard deviation 1.6×10^{-4} /day (corresponding to a mean half-life of 44 months).

The per cell rate at which latently infected cells reactivate was estimated to be log10-normally-distributed with mean -5 and standard deviation 0.8. This rate was calculated by first estimating the total number of cells exiting the latent reservoir per day based on the timing of rebound in studies of individuals who underwent monitored treatment interruption [1,13,14] (log10-normal with mean 1.7 and standard deviation 1.0), and combining it with estimates of the distribution of the frequency of latently infected cells by the intact proviral DNA assay (IDPA) in individuals on long-term suppressive ART initiative during chronic infection [15] (log10-normal with mean -4.3, corresponding to 50 per million resting CD4+ T cell, and standard deviation 0.6). The total body number of resting memory CD4+ T cells was estimated as $\sim10^{11}$, assuming ~1000 cells/uL in a blood volume of ~5L, of which ~half are resting memory, and that there are 50 times more CD4 cells in tissue compared to peripheral blood.

The growth rate of infection during rebound was taken from studies of early viral growth in acute infection [7,10] as 1.0 ± 0.4 /day (mean ± standard deviation). We assumed an average lifespan of productively infected cells as 1 day [16] and the average length of the eclipse phase as also 1 day [17]. Together these last three parameters determined a distribution of R_0 values centered on 4.0 (95% between 1.8-6.9). As in previous work we assumed that the variance in the number of secondary infections per cell is described by the variance-to-mean ratio ρ ("Fano factor"), which we take to be log10-normally distributed with mean 1 and standard deviation 0.5 (e.g. median value of 10). Together the mean (R_0) and variance in secondary infections determine the probability that a given viral lineage starting from a single reactivated cell will establish exponentially-growing infection (P_{Est}).

Inference

We use a Bayesian inference framework described previously [2] to predict the future probability of rebound (vs cure) in the London patient. We assume that viral dynamics in this patient are governed by our model but with an unknown number of remaining cells in the latent reservoir and with uncertainty in the viral dynamics parameters. At the time of ART stop, we use the observation of a negative quantitative viral outgrowth assay (QVOA) on 24 million resting CD4 T cells to inform the reservoir size [18]. We assume that for every 1 cell detected by QVOA there are a total of 50 latently infected cells that could potentially reactivate, based on the population level mean size of the reservoir by intact viral DNA assay (IDPA) [15]. We define "cure" as no rebound happening within 70 years.

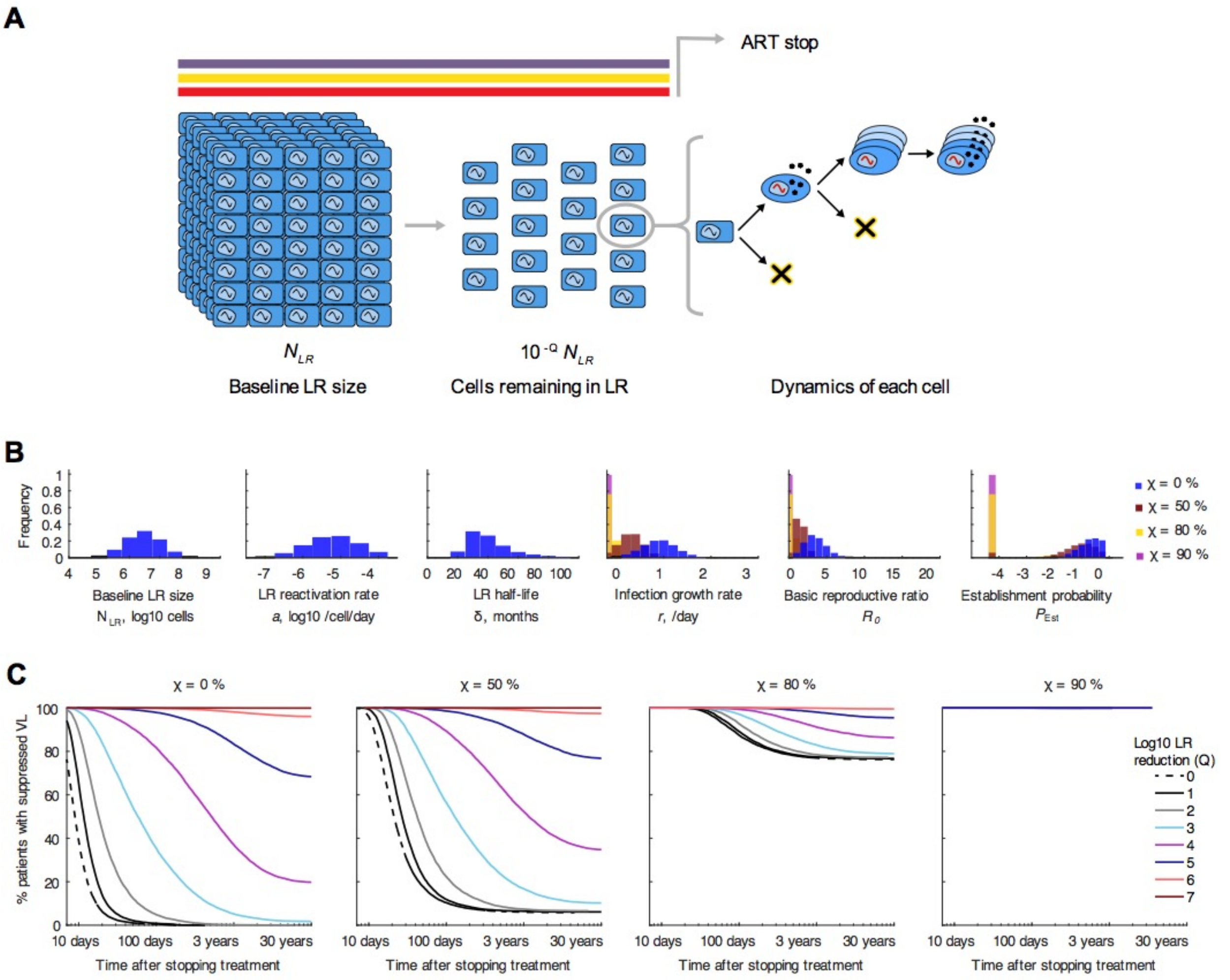

Supplementary Figure 1: Model of reservoir dynamics and viral rebound used to simulate outcomes as a function of reservoir size and chimerism. A) The model considers the dynamics of cells remaining in the latent reservoir at the time of ART stop. Results are shown as a function of

the reduction in the latent reservoir size (log10 reduction Q) from a typical baseline value (population mean 1 IUPM by QVOA or 50 IUPM by IDPA). The stochastic model tracks both latently infected resting CD4+ T cells (rectangles) and productively infected CD4+ T cells (ovals). Each arrow represents an event that occurs in the model. Latent cells can die or reactivate, and actively infected cells can die or produce virions that lead to newly infected cells, which pass through an eclipse phase before becoming productively infected. If CCR5Δ32/Δ32 chimerism (c) is non-zero, the number of new infections is reduced by 1- c. B) Population-level distributions of parameter values used in simulations. With increasing chimerism, the net viral fitness, described by the basic reproductive ratio, is reduced to R_0(1- c), which also leads to reductions in the infection growth rate and the probability a given lineage started by a single infected cell will establish. C) Model predictions shown as survival curves: the percentage of patients in a cohort who have not yet experienced viral rebound, plotted as a function of the time (logarithmic scale) after ART interruption. For very high chimerism, no rebound can occur.

References

1. Hill AL, Rosenbloom DIS, Fu F, Nowak MA, Siliciano RF. Predicting the outcomes of treatment to eradicate the latent reservoir for HIV-1. Proc Natl Acad Sci. 2014;111: 13475–13480. doi:10.1073/pnas.1406663111
2. Hill AL, Rosenbloom DIS, Goldstein E, Hanhauser E, Kuritzkes DR, Siliciano RF, et al. Real-Time Predictions of Reservoir Size and Rebound Time during Antiretroviral Therapy Interruption Trials for HIV. PLOS Pathog. 2016;12: e1005535. doi:10.1371/journal.ppat.1005535
3. Henrich TJ, Hatano H, Bacon O, Hogan LE, Rutishauser R, Hill A, et al. HIV-1 persistence following extremely early initiation of antiretroviral therapy (ART) during acute HIV-1 infection: An observational study. PLOS Med. 2017;14: e1002417. doi:10.1371/journal.pmed.1002417
4. Fine P, Eames K, Heymann DL. “Herd immunity”: a rough guide. Clin Infect Dis Off Publ Infect Dis Soc Am. 2011;52: 911–916. doi:10.1093/cid/cir007
5. Herz AVM, Bonhoeffer S, Anderson RM, May RM, Nowak MA. Viral dynamics in vivo: limitations on estimates of intracellular delay and virus decay. Proc Natl Acad Sci. 1996;93: 7247–7251.
6. Nowak MA, Lloyd AL, Vasquez GM, Wiltrout TA, Wahl LM, Bischofberger N, et al. Viral dynamics of primary viremia and antiretroviral therapy in simian immunodeficiency virus infection. J Virol. 1997;71: 7518–7525.
7. Ribeiro RM, Qin L, Chavez LL, Li D, Self SG, Perelson AS. Estimation of the Initial Viral Growth Rate and Basic Reproductive Number during Acute HIV-1 Infection. J Virol. 2010;84: 6096–6102. doi:10.1128/JVI.00127-10

8. Gupta RK, Abdul-Jawad S, McCoy LE, Mok HP, Peppa D, Salgado M, et al. HIV-1 remission following CCR5Δ32/Δ32 haematopoietic stem-cell transplantation. Nature. 2019;568: 244–248. doi:10.1038/s41586-019-1027-4
9. Henrich TJ, Hanhauser E, Marty FM, Sirignano MN, Keating S, Lee T-H, et al. Antiretroviral-Free HIV-1 Remission and Viral Rebound After Allogeneic Stem Cell Transplantation: Report of 2 Cases. Ann Intern Med. 2014;161: 319–327. doi:10.7326/M14-1027
10. Konrad BP, Taylor D, Conway JM, Ogilvie GS, Coombs D. On the duration of the period between exposure to HIV and detectable infection. Epidemics. 2017;20: 73–83. doi:10.1016/j.epidem.2017.03.002
11. Siliciano JD, Kajdas J, Finzi D, Quinn TC, Chadwick K, Margolick JB, et al. Long-term follow-up studies confirm the stability of the latent reservoir for HIV-1 in resting CD4+ T cells. Nat Med. 2003;9: 727–728. doi:10.1038/nm880
12. Crooks AM, Bateson R, Cope AB, Dahl NP, Griggs MK, Kuruc JD, et al. Precise Quantitation of the Latent HIV-1 Reservoir: Implications for Eradication Strategies. J Infect Dis. 2015; jiv218. doi:10.1093/infdis/jiv218
13. Ruiz L, Carcelain G, Martínez-Picado J, Frost S, Marfil S, Paredes R, et al. HIV dynamics and T-cell immunity after three structured treatment interruptions in chronic HIV-1 infection. AIDS. 2001;15: F19–F27.
14. Luo R, Piovoso MJ, Martinez-Picado J, Zurakowski R. HIV Model Parameter Estimates from Interruption Trial Data including Drug Efficacy and Reservoir Dynamics. PLoS ONE. 2012;7: e40198. doi:10.1371/journal.pone.0040198
15. Bruner KM, Wang Z, Simonetti FR, Bender AM, Kwon KJ, Sengupta S, et al. A quantitative approach for measuring the reservoir of latent HIV-1 proviruses. Nature. 2019;566: 120–125. doi:10.1038/s41586-019-0898-8
16. Markowitz M, Louie M, Hurley A, Sun E, Di Mascio M, Perelson AS, et al. A Novel Antiviral Intervention Results in More Accurate Assessment of Human Immunodeficiency Virus Type 1 Replication Dynamics and T-Cell Decay In Vivo. J Virol. 2003;77: 5037–5038. doi:10.1128/JVI.77.8.5037-5038.2003
17. Mohammadi P, Desfarges S, Bartha I, Joos B, Zangger N, Muñoz M, et al. 24 Hours in the Life of HIV-1 in a T Cell Line. PLOS Pathog. 2013;9: e1003161. doi:10.1371/journal.ppat.1003161
18. Rosenbloom DIS, Elliott O, Hill AL, Henrich TJ, Siliciano JM, Siliciano RF. Designing and Interpreting Limiting Dilution Assays: General Principles and Applications to the Latent Reservoir for Human Immunodeficiency Virus-1. Open Forum Infect Dis. 2015;2: ofv123. doi:10.1093/ofid/ofv123

Supplementary table: lymph node tissue analysis for HIV-1 DNA by ddPCR

Lymph node (27 months post ATI)						
	**Sample**	**Target**	**Replicates tested (n)**	**Cells tested (n)**	**Copies/million cells**	**Clinical interpretation***
**ddPCR**	Lymph node	LTR	14	2068220	33.6	Positive
	Water	LTR	4	0	-	
	PBMC	LTR	4	191290	<5.1	
	U1	LTR	4	3091	3409253	
	Lymph node	Integrase	8	1181840	<0.9	Negative
	Water	Integrase	2	0	-	
	PBMC	Integrase	2	97240	<10.3	
	U1	Integrase	2	1540	1720000	
	Lymph node	GAG	10	1349700	5.1	Positive
	Water	GAG	2	0	-	
	PBMC	GAG	2	96177	<10.4	
	U1	GAG	2	1613	1391197	
**IPDA**	Lymph node	PSI	14	2068220	1.6	Negative
	Water	PSI	4		-	
	PBMC	PSI	4	236720	<4.2	
	U1	PSI	4	2860	2289161	
	Lymph node	ENV	14	2068220	26.1	Positive
	Water	ENV	4	0	-	
	PBMC	ENV	4	236720	<4.2	
	U1	ENV	4	2860	2313986	
	**No double positive droplet in IPDA (PSI+ENV+) SHEARING INDEX 0.39**					

ATI = analytical treatment interruption
ddPCR = Digital Droplet PCR
IPDA = intact proviral DNA assay
* = based on ≤1 positive droplet per assay

Supplementary table: Peripheral CD4 T cell subset analysis for HIV-1 DNA by ddPCR

CD4$^+$ T-cells (28 months post ATI)						
	**Sample**	**Target**	**Replicates tested (n)**	**Cells tested (n)**	**Copies/million cells**	**Clinical interpretation***
**ddPCR**	CD4 cells	LTR	8	424160	<2.4	Negative
	T naive	LTR	8	282920	<3.5	Negative
	T memory	LTR	8	524920	6.7	Positive
	Water	LTR	4	0	-	
	PBMC	LTR	2	86790	<11.5	
	U1	LTR	2	1562	2887324	
**IPDA**	T memory	PSI	6	286770	21.5	Positive
	Water	PSI	6	0	-	
	PBMC	PSI	2	63690	<15.7	
	U1	PSI	2	3542	2335404	
	T memory	ENV	6	886380	6.9	Negative
	Water	ENV	6	0	-	
	PBMC	ENV	2	63690	<15.7	
	U1	ENV	2	3542	234826	
	**One double positive droplet in IPDA (PSI+ENV+) SHEARING INDEX 0.24**					

ATI = analytical treatment interruption
ddPCR = Digital Droplet PCR
IPDA = intact proviral DNA assay
* = based on ≤1 positive droplet per assay

Supplementary table: Gut tissue analysis for HIV-1 DNA by ddPCR

	Gut biopsies (22 months post ATI)					
	**Sample**	**Target**	**Replicates tested (n)**	**Cells tested (n)**	**Copies/million cells**	**Clinical interpretation***
**ddPCR**	Ileum	LTR	4	228580	6.7	Negative
	Caecum	LTR	4	358600	<2.8	Negative
	Rectum	LTR	4	151800	<6.6	Negative
	Water	LTR	2	0	-	
	PBMC	LTR	2	94050	<10.6	
	U1	LTR	2	1760	2800000	
	Ileum	Integrase	4	201080	<5.0	Negative
	Caecum	Integrase	4	326700	5.4	Negative
	Rectum	Integrase	4	136400	11.3	Negative
	Water	Integrase	2	0	-	
	PBMC	Integrase	2	97240	<10.3	
	U1	Integrase	2	1540	1720000	

ATI = analytical treatment interruption
ddPCR = Digital Droplet PCR
* = based on ≤1 positive droplet per assay

Supplementary table: HIV-1 DNA reservoir measurements across tissue sites by qPCR. Quantification is expressed in copies (c) HIV-1 DNA per million cells. ND: not done. IPDA: intact proviral DNA assay; qPCR quantitative polymerase chain reaction.

Tissue site And input	Sample month post ATI	LTR	Packaging region	Positive Wells	Env	Positive Wells
**Lymph node 75000 cells**	27	Negative <7.5 c/10^6 cells	76/10^6 cells (Input 250000)	2/20	70 c/10^6 cells (Input 250000)	4/20
**T. ileum 150000 cells**	22	Negative <7.5 c/10^6 cells	ND	-	ND	-
**Caecum 150000 cells**	22	Negative <7.5 c/10^6 cells	ND	-	ND	-
**Sigmoid 112500**	22	Negative <7.5 c/10^6 cells	ND	-	ND	-
**Rectum 150000 cells**	22	Negative <7.5 c/10^6 cells	ND	-	ND	-

Supplementary table: results of western blotting of plasma at various times post allo-HSCT.

Days post allo-HSCT	Observed Bands	Bands left
26	GP160, GP110/120, GP41, p68, p55, p52, p40, p31, p24, p18	None
423	GP160, **GP110/120 faint** , GP41, **p31 faint**, **p24 very faint**	p68, p55, p52, p40, p18
732	GP160, **GP110/120 faint** , GP41	p68, p55, p52, p40,P24, p18, P31
811	GP160, **GP110/120 faint**, GP41, **p31 very faint**	p68, p55, p52, p40, p24, p18
965	GP160, **GP110/120 very faint,** GP41, P18	p68, p55, p52, p40, p24, p31
1044	GP160, **GP110/120 very faint**	GP41, p68, p55, p52, p40, p31, p24, p18
1162	GP160, **GP110/120 very faint**	GP41, p68, p55, p52, p40, p31, p24, p18
1191	GP160, **GP110/120 very faint**	GP41, p68, p55, p52, p40, p31, p24, p18
1310	GP160, **GP110/120 very faint**	GP41, p68, p55, p52, p40, p31, p24, p18
